# Effects of Time-Restricted Feeding on Energy Balance: A Cross-Over Trial in Healthy Subjects

**DOI:** 10.3389/fendo.2022.870054

**Published:** 2022-04-27

**Authors:** Riqiang Bao, Yingkai Sun, Yiran Jiang, Lei Ye, Jie Hong, Weiqing Wang

**Affiliations:** ^1^Department of Endocrine and Metabolic Diseases, Shanghai Institute of Endocrine and Metabolic Diseases, Ruijin Hospital, Shanghai Jiaotong University, School of Medicine, Shanghai, China; ^2^Shanghai National Clinical Research Center for Metabolic Diseases, Key Laboratory for Endocrine and Metabolic Diseases of the National Health Commission of the PR China, Shanghai National Center for Translational Medicine, Ruijin Hospital, Shanghai Jiaotong University, School of Medicine, Shanghai, China

**Keywords:** time-restricted feeding, energy balance, energy excretion, energy expenditure, blood glucose (BG)

## Abstract

Time-restricted feeding (TRF) has been recently reported as an effective dietary intervention for losing body weight, implying a negative energy balance, without restricting nutrient intake. However, the detailed energy balance alteration caused by TRF remains unclear. This study was a randomized controlled clinical trial using a within-subject cross-over design. Twelve healthy, normal-weighted volunteers (age: 24 ± 2.3 years; BMI: 21.9 ± 1.71 kg/m^2^; 7 females) were studied under a rigorous control for calorie intakes, physical activities as well as sleep-wake cycle to evaluate the energy balance systematically. Each participant consumed an isocaloric diet within either a 5.5-hour TRF or 11-hour control schedule. All energy intake and excretion were traced and collected and accessed by bomb calorimetry. Energy expenditure and substrates oxidation were monitored in a metabolic chamber. TRF compared with control schedule is associated with a 22.7% increase in fecal energy loss (Δ = 32.25 ± 9.33 Kcal, p = 0.005) and a trend in increasing 14.5% urine energy loss (Δ = 6.67 ± 3.14 Kcal, p = 0.058) without change energy expenditure. In total, a negative energy balance (Δ = -45.95 ± 19.00 Kcal, p = 0.034), which was equal to -2.6% of total energy intake, has been observed during TRF interventions. In the meantime, glycemic profiles, heart rate, respiration rate as well as metabolic flexibility were also improved during TRF intervention. Taken together, our findings unravel the mystery of how TRF regulates energy balance, supporting the use of TRF as an alternative dietary strategy for weight loss.

## Introduction

During the past few decades, the prevalence of obesity and related chronic diseases has increased dramatically (1). The current first line of therapy for overweight/obesity is aggressive lifestyle modifications including reducing calorie intake, improving diet quality, and increasing physical activity (2, 3). However, long-term adherence and maintenance to such strategies are challenging (4). Time-restricted feeding (TRF) is a specific intermittent fasting protocol that has gained attention as a more acceptable and feasible regimen. Compared to the traditional calories restricted diets, TRF encourages individuals to consume their usual diet within a limited eating window without necessarily altering diet quantity or quality per se (5). Both cross-over and parallel-arms studies in humans have confirmed the modest reductions in body weight and fat mass after TRF intervention (6–13). TRF was also reported to improve glycemic and lipid profiles, reduced plasma triglycerides, inflammatory markers (10, 14, 15) and other metabolic parameters, including blood pressure, oxidative stress, and sleep quality (11, 16, 17).

Body weight changes are fundamentally based on an imbalance between energy intake and energy expenditure over a certain period of time (18, 19). Experiments in rodents have shown that TRF within an 8-hour period increases 24-hour energy expenditure. But this phenomenon has not been validated yet in human beings (20). Some studies speculated that TRF-induced 1-4% weight loss over a short duration (1-16weeks) may be caused by reduced energy intake. During the experiment, participants were allowed for ad libitum intake. Nevertheless, confining the period of eating window might reduce energy intake by ~350–500 kcal/day (8, 9, 11, 21–24). However, the limitation is that food intake measurement in these studies was either based on participants’ self-declarations or left untracked (25). Compared with ad libitum intake, several isocaloric intake studies offer different perspectives. In these studies, the subjects consumed an isocaloric diet under rigorous control conditions, but their results are quite different. While both body weight and fat mass were decreased after 8 weeks or 4 weeks of TRF intervention, as reported by (10, 26) respectively, other studies have found no significant body weight change in TRF group compared with the control group (14, 27). The studies to date are limited, there is still controversy about how TRF regulates energy balance and thus affects body weight.

In this study, we performed a randomized within-subject cross-over trial to systematically evaluate the energy balance of TRF under rigorous control for calorie intakes, physical activities as well as the sleep-wake cycle. The primary aim of this study was to compare the energy balance of TRF (5.5-hour eating period) and control (11-hour eating period) schedules in healthy subjects. To systematically explain how TRF creates a negative energy balance without reducing calorie intakes. Secondary aims included the acute effects of TRF on blood glucose and physiological parameters.

## Materials and Methods

### Ethical Approval

**Figure 1:**
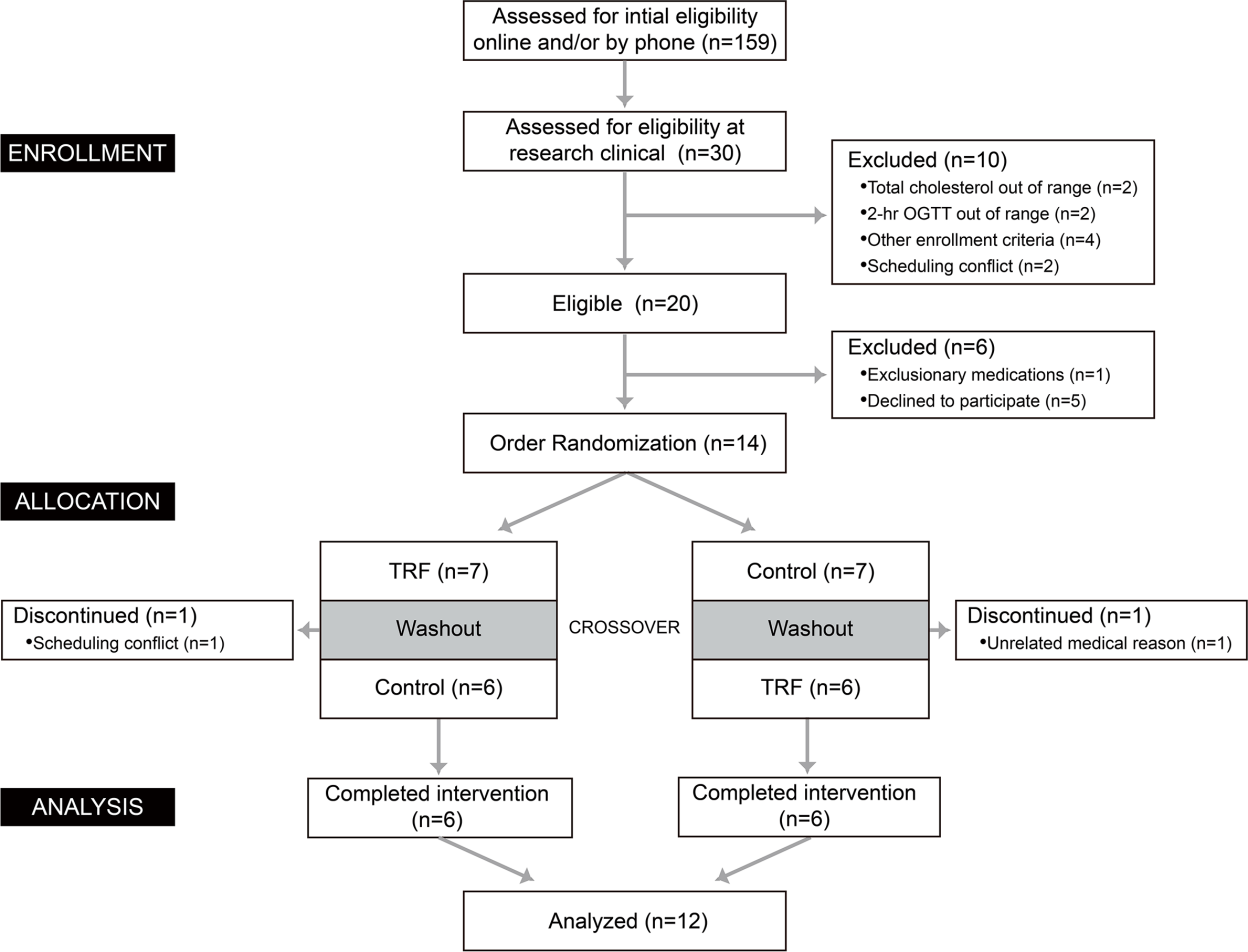
CONSORT flow diagram indicating the design of the trial.

This study protocol was approved by the ethics committee of Ruijin Hospital affiliated to Shanghai Jiaotong University (Ref. No. 2019-246). Clinical trial registered as “The Impact of Time-Restricted Feeding (TRF) on Human Energy Metabolism” at chictr.org.cn as ChiCTR2000038421. A CONSORT flow diagram outlining the study protocol is displayed in **Figure 1**.

### Participants

Fourteen healthy participants were recruited from 159 subjects who were interested in this study and submitted our lifestyle questionnaire online. Finally, twelve participants completed this study. The entry criteria included BMI ≤ 25 kg/m^2^, Hb1Ac between 4.7 and 6.4%, fasting glucose between 70 and 110 mg/dl, normal lipid profiles and thyroid parameters, and steady eating habits with breakfast between 7:00 and 9:00, lunch between 11:00 and 14:00, and dinner between 18:00 and 20:00. We excluded subjects with extensive change of body weight (± 3%) and medication use over the past 3 months, diarrhea, diabetes, eating disorder, psychiatric disorder, breastfeeding or pregnant as well as time-shifted works. All participants provided written informed consent prior to participation in the trial.

### Study Design

The total design of this study was exhibited in **Supplementary Figure 1A**. Each eligible subject underwent a cross-over dietary intervention, in which participants were treated with TRF (eat within 5.5 hours: first meal at 8:00, second meal at 10:45, and third meal at 13:30) and control (eat within 11 hours: first meal at 8:00, second meal at 13:30, and third meal at 19:00) treatment. The order of two dietary schedules for each individual was randomized with a one-week washout period. Each dietary intervention was confined to the metabolic chamber for 3 days, during which calories of each meal and physical activities were rigorously controlled. To exclude the compounding effect of food type, an artificial meal containing 55% carbohydrates, 30% fat, and 15% protein was provided. Participants underwent standardized acclimatization on the first day before the conduction of two diet interventions on the second day. Then all participants were asked to maintain the same lifestyle on the third day until all feces sourced from day 2 intervening food were completely excreted. During the first day and third day, all the food provided was labeled with carmine dye. During the second day, all food was labeled with brilliant green dye to trace and collect the excretion of feces from this day. Individual daily energy requirements were calculated based on body weight, height, and age, using the Harris-Benedict equation with a fixed physical active level (PAL) of 1.2, corresponding to low physical activity (28). Each meal was consumed at a constant rate within 30 minutes during the intervention. Daily fluid intake was provided with the standard of 30ml/kg body weight. Daily activities during the intervention were strictly controlled according to the standardized schedule (**Supplementary Figure 1B**). During the unconstrained hours, participants were only allowed to engage in mild activities including watching videos, using computers, walking in the chamber. To meet the PAL of 1.2, participants were asked to do 15 minutes of exercise on their 40% VO_2max_ level by using an ergometer 3 times every day. During the washout days, participants were asked to keep a stable lifestyle, maintain the diet habit as well as avoid intensive exercise, and the body-weight changes were controlled within 1%. Daytime was defined as 8:00 to 20:00 and nighttime was defined as 20:00 to 8:00 of the next day.

### Calorie and Macronutrients in Food, Feces, and Urine

All food and feces labeled with brilliant green dye (used in the intervention) were collected, mixed well, and lyophilized. 24-hour urine from the intervention day was collected and underwent direct lyophilization. Lyophilization was performed at -50°C using an instrument (SJIA-5FE, ShuangJia Instrument, Ningbo, China). Calories were measured by using bomb calorimetry according to the published protocol (29) (Parr 6400 Calorimeter, PARR Instrument Co., Moline, IL). To minimize the error of measurements, all samples were measured repeatedly. If the difference between repeated samples > 50 Cal, measurements were repeated until the difference < 50 cal. We averaged two qualified measurements as the final calories of each sample. Food and fecal protein concentration were determined by the Kjeldahl method, fat concentration was determined by the Soxhlet method, and carbohydrates concentration was calculated by subtracting the protein, fat, water, and ash from the total weight of the sample.

### Energy Expenditure and Substrates Oxidation

To measure the substrate oxidation and energy expenditure, participants resided in the metabolic chamber for the whole experiment period. The metabolic chamber is an airtight room with a volume of 30,000L each (Fuji Medical Science Co. Ltd., Chiba, Japan). The chamber is furnished with an adjustable bed, desk, chair, bicycle ergometer, wash basin, and toilet. Air in the chamber was pumped out at a rate of 70 L/min. Room temperature and relative humidity were maintained at 25.0 ± 0.1°C and 50.0 ± 1.5%, respectively. Concentrations of oxygen (O2) and carbon dioxide (CO2) in the sample air were analyzed using an online process mass spectrometer (Prima PRO, Thermo Fisher Scientific, Cheshire, UK) after dehydration. The mass spectrometry was calibrated monthly using standard gas and the accuracy for O2 and CO2 is 0.0013%. O2 consumption (VO2) and CO2 production (VCO2) were calculated by Henning method (30). To minimize the error of the metabolic chamber, we calibrated the accuracy by alcohol combustion. The precision of metabolic chamber was 99.2 ± 0.9% for O2 consumption and 100.0 ± 1.1% for CO2 production during the study. VO2 and VCO2 data produced by Henning’s method were obtained at the frequency of 1 min. And then we aggregated these minute-based data into hourly-based for statistical comparison. Macronutrient oxidation and energy expenditure were calculated using the Weir equation with urinary nitrogen excretion (31). To correct the measured RQ for protein oxidation, nonprotein RQ (npRQ) was calculated by using nitrogen excretion in 24-h urine. Twenty-four-hour thermic effect of food (TEF) was determined by plotting energy expenditure against physical activity level, and the intercept of the regression line at the lowest physical activity represents SMR and TEF. All women performed respiratory chamber testing during the follicular or luteal phases of their menstrual cycle.

### Continuous Glucose Monitoring

All participants were fitted with a CGM sensor and transmitter (Dexcom G5, Dexcom, San Diego, CA, USA) during the study period. The glucose-oxidase-based electrochemical sensor was inserted into subcutaneous tissue of the abdomen, followed by an initial warm-up period for 2 hours after sensor insertion. Sensor insertion began the day before the first acclimatization day and there was two days of calibration before data collection during the dietary intervention. According to company recommendations, the sensors were calibrated at least once every 12 hours by finger prick (FreeStyle Lite; Abbott Laboratories, Abbott Park, IL). After a washout period, participants were asked to insert a new sensor the day before the study.

### Maximal Oxygen Uptake

Prior to the trial, each participant performed a maximal oxygen uptake (VO_2max_) test on a cycle ergometer (939E, Monark Ltd, Vansbro, Sweden). Oxygen consumption and carbon dioxide production were measured by a breath-by-breath portable gas analyzer (K5, COSMED, Rome, Italy). The test procedure started with 5 minutes warm-up at a workload of 0 W. Then, the workload was increased by 25W or 20W every minute for men or women, respectively, while participants maintained a pedaling rate of 60 rpm until exhaustion despite verbal encouragement. Participants met at least 2 of the following criteria were considered maximal: respiratory exchange ratio > 1.15, maximal heart rate > 90% of the predicted max (220-age), perceived exertion (RPE) ≥ 18, a plateau in VO2 despite an increasing workload. The exercise intensity was set at 40% VO_2max_ (light intensity) in this study.

### Physiological Parameters Measure

Noninvasive blood pressure, 3-lead ECG, and peripheral pulse oximetry SpO2 were continuously monitored by a Cardiac Telemetry System (WEP-5204C, Nihon Kohden Co., Tokyo, Japan). Systolic and diastolic blood pressure were measured every 10 minutes during the study. Heart rate, breath rate, and SpO2 were measured every second during the study. Equivital LifeMonitor system (EQ02 LifeMonitor, Hidalgo Ltd, Cambridge, UK), capable of logging physiological data including respiratory inductance plethysmography, posture, activity, and skin temperature every 15 seconds was used during study. Participants were fitted with a correctly sized chest vest dependent on their chest circumference. Axillary skin temperature was measured by an infrared sensor. Ambulation status was measured by accelerometer sensor and divided into different levels: stationary, moving slowly, and moving fast. Bioelectrical impedance analysis (Inbody 770, Inbody Co. Ltd., Seoul, Korea) was used to measure body composition and weight. Height was measured in centimeters using a height measurement instrument (RGZ-120, DongFang Scales Co. Ltd., Shanghai, China).

### Biochemical Analysis

Blood sampling was performed at 0h before and 0.5h, 1h, and 2h after every three meals during the intervention. Samples were collected by using a standard venipuncture. Plasma and serum were centrifugated at 4°C and stored at -80°C until analysis. Insulin was measured by an autoanalyzer (ARCHITECT ci16200 analyzer, Abbott Laboratories, USA).

### Appetite and Sleepiness by VAS

Participants rated their hunger, fullness, stomach fullness, desire to eat, capacity to eat, and sleepiness using Visual Analog Scales (VAS; a 0-100 mm scale). The higher the scale, the stronger the sensation. VAS surveys were administered for total fourteen times at pre-meal (0h), 0.5, 1, and 2 hours post meal, 10:00 pm, and the next day morning at 7:00 am.

### Statistical Analysis

Based on our preliminary data and a previous report (29), we assumed that the 24-hour calories excretion is 6.7 ± 1.2% of intake during control dietary schedule and 8.6 ± 2.7% of intake during TRF. We calculated that 12 participants in each group would be needed to give this study 82% power to detect 1.9% difference of calories excretion in feces at a two-side alpha value of 0.05.

Participants were randomly allocated in 1:1 ratio to 5.5-hour TRF group or 11-hour control group. After the washout period, crossover was carried out for both groups. The generation of allocation sequence was based on the random-number table.

Statistical analyses were performed using R Studio (version 3.6.0, Boston, Massachusetts). Since the sample size of this study is comparatively small, we exert pairwise t-test with Holm–Bonferroni adjustment to reduce the sampling error. A two-tailed p value of ≤ 0.05 was considered as statistically significant. The Shapiro-Wilk test was used to check the data distribution. For parameters with non-Gaussian distribution, the Mann-Whitney test was used. For parameters with normal Gaussian distribution, a two-tailed pairwise t-test was applied. When applicable, non-parametric Wilcoxon matched-pairs signed ranks test was used. To analysis the time course data, two-way repeated measures ANOVA models were used to evaluated the interaction between time and groups on dependent variable. Baseline data are reported as mean ± standard deviation (SD), and other data are presented as mean ± standard error of the mean (SEM). AUCs were calculated by the trapezoidal rule. To evaluate the contribution of insulin and carbohydrate oxidation in explaining TRF-induced change of postprandial glycemic response, a linear mixed-effects model with incremental glucose AUC as the dependent variable, diet intervention (control as the reference) and pre-prandial glucose as independent variable, participants and meals as random effect was constructed, and pre-prandial insulin and postprandial carbohydrate oxidation were added as independent variable to observed the changes of each effect.

## Results

### Participants

Fourteen participants were included and twelve (5 men and 7 women) of them completed this study (**Figure 1**). Each participant underwent a cross­over trial, in which participants were treated with TRF (eating period: 8:00 AM to 13:30 AM) and control (eating period: 8:00 AM to 19:00 AM) dietary schedules. The order of two dietary schedules for each individual was randomized and the two interventions were separated by a one-week washout period. During the washout period, participants kept their usual lifestyle and the mean body weight upon entering the chamber were same (60.65 ± 2.80 kg versus 60.70 ± 2.77 kg, Δ = 0.05 ± 0.12 kg, p = 0.988). The baseline characteristics of participants were measured from 6:00 to 7:00 in the morning of the intervening day (**Table 1**), during which participants maintained supine position without other activities. Neither biochemical nor physiological profiles were significantly different between groups.

**Table 1:** Basal characteristics of participants and pre-intervention parameters.

	Control	TRE	p Value
**Participants characteristics**			
N	12	12	–
Age (Year)	24 ± 2.3	–	–
BMI (Kg/m^2^)	21.9 ± 1.71	–	–
HbA1c (%)	5.11 ± 0.19	–	–
Fasting glucose (mg/dl)	91.44 ± 6.66	–	–
Fasting insulin (μIU/mL)	7.63 ± 3.96	–	–
Triglyceride (mmol/L)	0.81 ± 0.19	–	–
Total cholesterol (mmol/L)	4.86 ± 0.79	–	–
LDL (mmol/L)	2.96 ± 0.76	–	–
HDL (mmol/L)	1.62 ± 0.27	–	–
**Pre-intervention parameters**			
Blood glucose (mg/dL)	92.97 ± 10.23	91.53 ± 7.76	0.703
Basal metabolic rate (Cal/Kg)	11.94 ± 2.28	11.06 ± 2.29	0.36
Respiratory quotient	0.84 ± 0.06	0.85 ± 0.06	0.724
Resting heart rate (BMP)	61.56 ± 8.79	61.13 ± 7.04	0.896
Resting respiratory rate (RPM)	17.41 ± 1.85	16.82 ± 1.62	0.418
Skin temperature (°C)	36.69 ± 0.85	36.62 ± 0.59	0.834
Systolic BP (mmHg)	102.21 ± 5.75	103.54 ± 7.8	0.639
Diastolic BP (mmHg)	61.83 ± 6.85	63.82 ± 6.07	0.46
Oxygen saturation (%)	98.26 ± 0.7	98.36 ± 0.9	0.765

*Data were presented as mean ± SD.

*Differences between group were tested by pairwise t-test with Holm–Bonferroni adjustment

*Pre-intervention parameters were collected from 6:00 to 7:00 in the morning of intervening day, during which participants maintained supine position without other activities.

### Energy Intake

Participants were designed to receive an isocaloric, controlled-nutrient food containing 55% carbohydrate, 30% fat, and 15% protein throughout the study. We confirmed the food energy density and macronutrients components by bomb calorimetry and chemical method, respectively. The energy density (Δ = 0.02 ± 0.05 Kcal/g, p = 0.666) and macronutrients are stable and equal except for labeled with brilliant blue or carmine dyes (**Supplementary Table 1**). As shown in **Table 2**, no significant differences in total energy, carbohydrate, fat, and protein intake were observed. Meanwhile, the intake time of three meals also showed no statistical difference. Any beverages containing energy were prohibited, and only mineral water was provided based on 30ml/kg body weight. Fluid intake volumes were same among two groups.

**Table 2:** Energy intake during 24-Hour TRF versus control intervention.

	Control	TRF	Percent change	p Value
**Energy intake**				
Total energy (Kcal)	1807.07 ± 67.95	1806.05 ± 67.16	0%	0.992
Carbohydrate (Kcal)	1045.61 ± 39.62	1045.01 ± 39.16	0%	0.992
Fat (Kcal)	520.06 ± 19.35	519.76 ± 19.13	0%	0.991
Protein (Kcal)	241.41 ± 8.98	241.27 ± 8.88	0%	0.991
**Intake time**				
First meal (min)	26.25 ± 1.40	25.83 ± 1.52	-2%	0.842
Second meal (min)	25.58 ± 1.29	26.67 ± 1.59	4%	0.603
Third meal (min)	25.67 ± 1.16	24.50 ± 1.41	-5%	0.530
**Fluid intake**				
Fluid volume (ml)	2023.09 ± 95.51	2064.50 ± 102.54	2%	0.770

*Data were presented as mean ± SEM.

*Differences between group were tested by pairwise t-test with Holm–Bonferroni adjustment.

*The percentage change was calculated by dividing the differences of mean between TRF and control groups.

### Energy Expenditure

**Figure 2:**
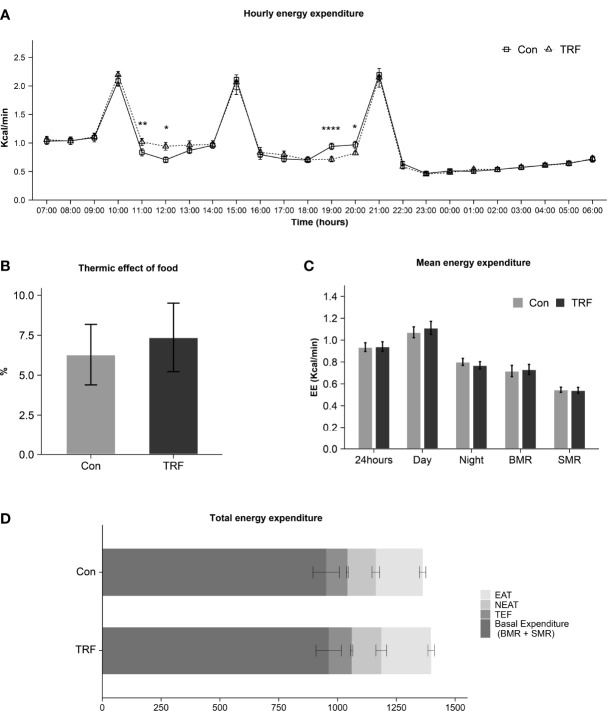
Hourly energy expenditure **(A)**, thermic effect of food **(B)**, mean energy expenditure **(C)**, and total energy expenditure **(D)** between control and TRF groups. Data are mean ± SEM, *P <0.05, **P<0.01, ****P≤ 0.0001.

In **Figure 2A** there is an overview of hourly energy expenditure for two groups. There was a statistically different interaction between time and groups on energy expenditure (F(23,253) = 2.804, p<0.001, **Supplementary Table 2**). Compared with control group, TRF slightly increased energy expenditure after second meal for 2 hours from 11:00 (Δ = 0.182 ± 0.055 Kcal/min, p = 0.007) to 12:00 (Δ = 0.237 ± 0.080 Kcal/min, p = 0.013). In contrast, after the third meal in control group, energy expenditure was also slightly increased for 2 hours from 19:00 (Δ = 0.226 ± 0.037 Kcal/min, p < 0.0001) to 20:00 (Δ = 0.147 ± 0.059 Kcal/min, p = 0.031). These differences were mainly attributed to postprandial TEF. But no significant difference was found in total TEF (Δ = 1.08 ± 3.45 %, p = 0.760) after reciprocal compensation (**Figure 2B**). The 24-hour mean energy expenditure (Δ = 0.005 ± 0.015 Kcal/min, p = 0.763), daytime mean energy expenditure (Δ = 0.040 ± 0.030 Kcal/min, p = 0.211), nighttime mean energy expenditure (Δ = -0.031 ± 0.019 Kcal/min, p = 0.129), basal metabolic rate (BMR) (Δ =0.015 ± 0.034 Kcal/min, p = 0.690), and sleeping metabolic rate (SMR) (Δ = -0.005 ± 0.021 Kcal/min, p = 0.826) were not significantly affected by TRF (**Figure 2C**). Furthermore, the exercise activity thermogenesis (EAT) (p = 0.223) as well as non-exercise activity thermogenesis (NEAT) (p = 0.827) were also not affected (**Figure 2D**).

### Energy Balance

**Figure 3:**
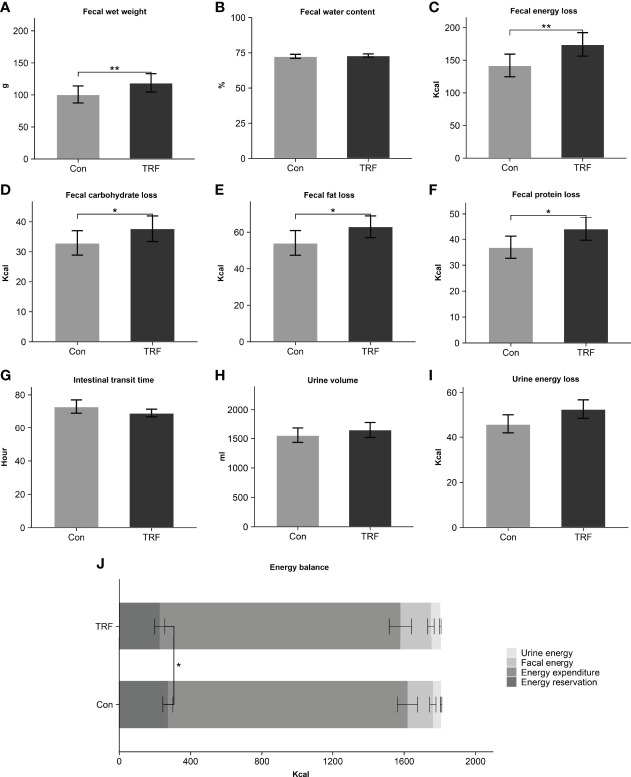
Fecal wet weight **(A)**, fecal water content **(B)**, fecal energy loss **(C)**, fecal carbohydrate loss **(D)**, fecal fat loss **(E)**, fecal protein loss **(F)**, intestinal transit time **(G)**, urine volume **(H)**, urine energy loss **(I)**, and energy balance **(J)** between control and TRF groups. Data are mean ± SEM, *P <0.05, **P<0.01.

As shown in previous results, the energy intake and expenditure showed no difference between groups and then we examined a third part, as an often-neglected factor, fecal energy loss. Surprisingly, after TRF intervention, total fecal wet weight (Δ = 18.14 ± 5.02 g, p = 0.004) was higher than control group while the percentage of water (Δ = 0.58 ± 1.02%, p = 0.579) was unaffected (**Figure 3**). The absolute number of calories lost in TRF group was significantly increased by 22.7% (Δ = 32.25 ± 9.33 Kcal, 174.28 ± 18.04 Kcal versus 142.03 ± 17.33 Kcal, p = 0.005) compared to control group. Macronutrients of feces including carbohydrate, fat, and protein (all p ≤ 0.047) were also increased accordingly. The transit time (Δ = -3.86 ± 4.14 hours, p = 0.371), as interval between the first meal labeled with brilliant blue and the last feces discharging with the same dye marker, was unaffected during TRF intervention. The change in the total volume of urine (Δ = -52.6 ± 91.01 ml, p = 0.575) between TRF and control group was not detected. However, calories in urine showed 14.5% (Δ = 6.67 ± 3.14 Kcal, p = 0.058) elevation in TRF group that nearly reached statistical significance. In aggregate, **Figure 3J** shows the energy balance status of both groups. Compared with control group, TRF intervention induced a lower energy balance level (226.69 ± 28.56 Kcal versus 272.64 ± 28.16 Kcal, Δ = -45.95 ± 19.00 Kcal, p = 0.034), mainly as a result of increased fecal energy losses.

### Substrate Oxidation

**Figure 4:**
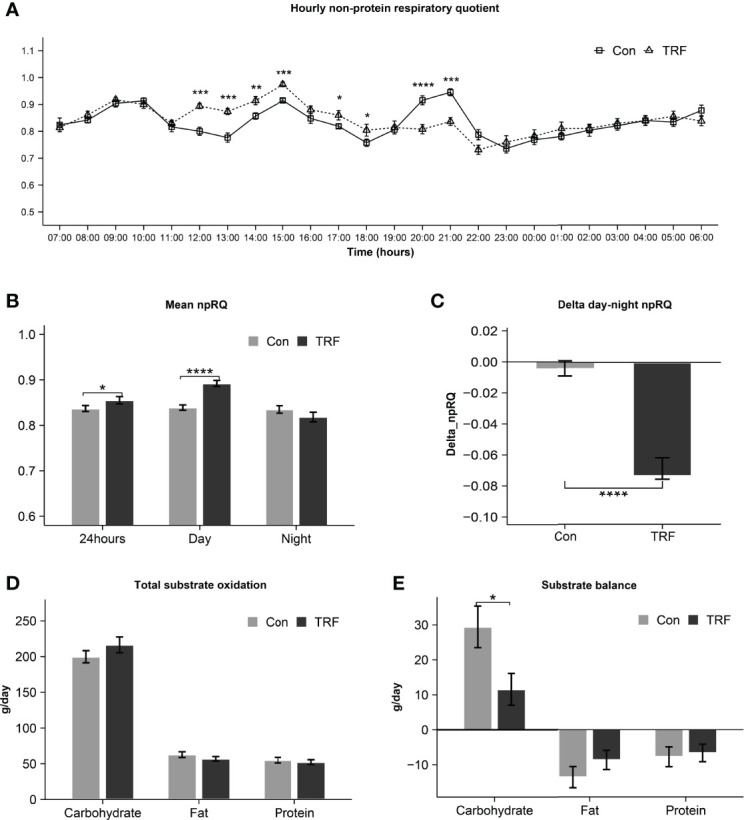
Hourly npRQ **(A)**, mean npRQ **(B)**, delta day-night npRQ **(C)**, total substrate oxidation **(D)**, and substrate balance **(E)** between control and TRF groups. Data are mean ± SEM, *P <0.05, **P<0.01, ***P<0.001, ****P≤0.0001.

An overview of hourly npRQ was shown in **Figure 4A**, there was a statistically different interaction between time and groups on RQ (F(23,253) = 6.603, p<0.001, **Supplementary Table 2**). Compared with control group, TRF increased npRQ nearly continuously across a 7-hour period from 12:00 to 18:00, except for 16:00 (all p ≤ 0.046). In contrast, control group increased npRQ after the third meal for only 2-hour period from 20:00 to 21:00 (all p < 0.001). As shown in **Figures 4B, C**, 24-hour mean npRQ (Δ = 0.018 ± 0.007, p = 0.030) and diurnal mean npRQ (Δ = 0.053 ± 0.008, p < 0.0001) were increased in TRF group. Nocturnal mean npRQ (Δ = -0.017 ± 0.008, p = 0.073) was marginally decreased in TRF group. The day to night transition, as npRQ was higher at daytime and have a lower trend at nighttime in TRF, which can be consider as a marker of metabolic flexibility (Δ = -0.070 ± 0.007, p < 0.001), was significantly increased when compared with control group. The 24-hour net oxidation of carbohydrate (Δ = 16.60 ± 8.32 g/day, p = 0.071) was marginally increased during TRF intervention as compared to control group, and fat oxidation (Δ = -5.87 ± 2.85 g/day, p = 0.064) was tended to decrease in TRF group. No statistical difference was observed in protein oxidation (Δ = -2.77 ± 1.86 g/day, p = 0.165) between groups. After correction for macronutrients intake and lost in feces, carbohydrate balance was significantly decreased in TRF group, as residual carbohydrate was 17.88g ± 8.13 g/day (p = 0.050) less than control group. But no differences were observed in either fat balance (Δ = 4.89 ± 2.92 g/day, p = 0.122) or protein balance (Δ =1.08 ± 1.84 g/day, p = 0.567, **Figures 4D, E**).

### Biochemical and Physiological Parameters

**Figure 5:**
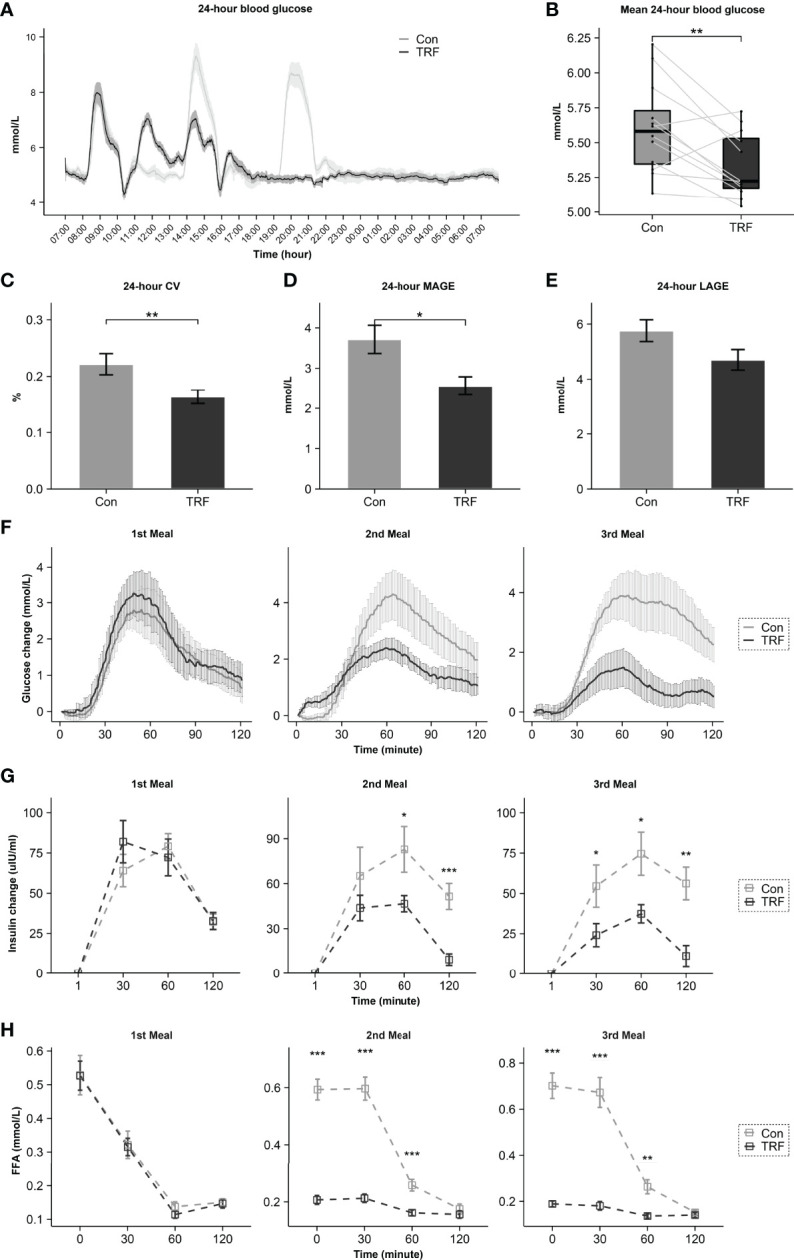
The effect of TRF on 24-hour CGM data **(A)**. Comparison of 24-hour mean glucose **(B)**, CV **(C)**, MAGE **(D)**, LAGE **(E)**, postprandial glycemic **(F)**, postprandial insulin **(G)**, and postprandial FFA **(H)** between control and TRF groups. Data are mean ± SEM, *P <0.05, **P<0.01, ***P<0.001.

**Figure 6:**
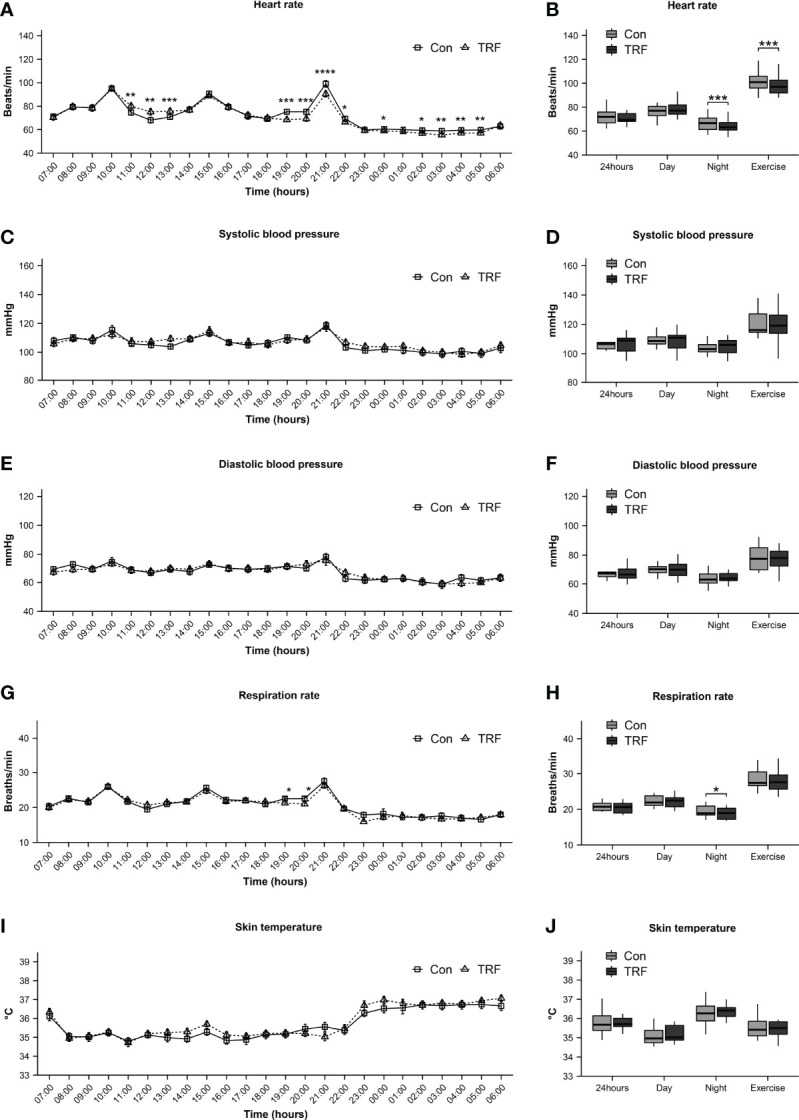
Heart rate **(A, B)**, systolic blood pressure **(C, D)**, diastolic blood pressure **(E, F)**, respiration rate **(G, H)**, and skin temperature **(I, J)** between TRF and control group. Data are mean ± SEM, *P <0.05, **P<0.01, ***P<0.001,****P≤0.0001.

CGM data revealed that 24-hour average blood glucose was significantly lower during TRF treatment as compared to control (Δ = -0.27 ± 0.05 mmol/L, p = 0.007, **Figures 5A, B**), which were mainly contributed by a significant reduction of diurnal blood glucose (Δ = -0.33 ± 0.06 mmol/L, p = 0.001), whereas nocturnal glucose level exhibited no statistical difference between groups (**Supplementary Table 3**). Glycemic variability was significantly blunted during TRF intervention, as reflected by the significantly lower coefficient of variation (CV) (Δ = -0.06 ± 0.01%, p=0.007) and mean amplitude of glycemic excursions (MAGE) (Δ = -1.16 ± 0.08 mmol/L, p=0.014, **Figures 5C**–**E**). Postprandial glycemic and insulinogenic responses to the two time-shifted meals (the 2nd and 3rd meals) were both significantly attenuated in TRF group as compared to control. The incremental area under curves of postprandial glucose and insulin were paralleling decreased (**Figures 5F, G** and **Supplementary Table 4**). Our mixed-linear model revealed that such extensive reduction of glycemic response to food during TRF could be partially explained by its pre-prandial glucose and insulin level (Pre-prandial glucose: p < 0.001; Pre-prandial insulin: p = 0.015), but no contribution from carbohydrate oxidation was observed (P = 0.207). There is still a large proportion of TRF-induced hypoglycemic effect that could not be explained by current data (P = 0.002, **Supplementary Table 5**). Pre- and post-prandial free fatty acid (FFA) were measured simultaneously with insulin and intravenous blood glucose. While no statistical differences of FFA between groups were observed around the 1st meal, both pre- and post-prandial FFA were substantially reduced during TRF (**Figure 5H** and **Supplementary Table 6**). There were a statistically different interactions between time and groups on heart rate (F(23,230) = 11,109, p < 0.001) and respiratory rate (F(23, 230) = 1.906, p = 0.009, **Supplementary Table 7**). As compared to control, heart rate was significantly higher for 3-hour period from 11:00 to 13:00 (all p ≤ 0.001) during TRF intervention. But continuously lower for almost 11-hour period from 19:00 to 05:00 (all p ≤ 0.038), except for 23:00 and 01:00 (**Figure 6A**). As a result, mean heart rate during the nighttime (Δ = -2.93 ± 0.59 beats/min, p < 0.001) and exercise (Δ = -4.07 ± 0.88 beats/min, p < 0.001) was significantly lower in TRF (**Figure 6B**). Nocturnal respiration rate (Δ = -0.58 ± 0.26 breaths/min, p=0.042) was slightly reduced in TRF group (**Figures 6G, H**). However, there were no significant interaction between time and group on either blood pressure or skin temperature (**Supplementary Table 7**). Neither hourly nor 24h-average data in blood pressure and skin temperature alternation were observed (**Figures 6C**–**F, I, J**).

### Appetite and Sleepiness Level by VAS

**Figure 7:**
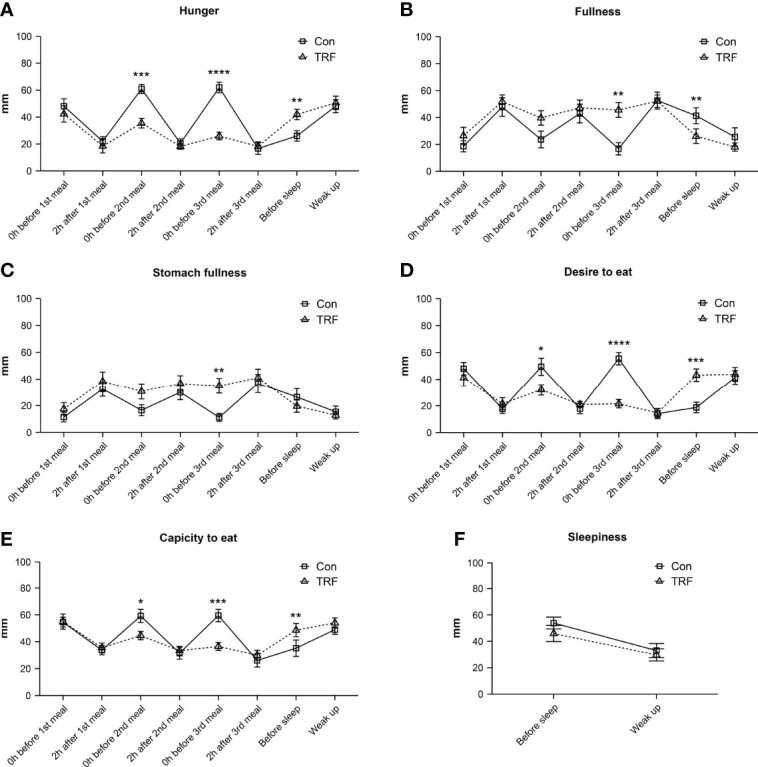
Subjective hunger **(A)**, fullness **(B)**, stomach fullness **(C)**, desire to eat **(D)**, capacity to eat **(E)**, and sleepiness **(F)** responses to VAS from participants during TRF and control intervention. Data are mean ± SEM, *P <0.05, **P<0.01, ***P<0.001,****P≤0.0001.

As shown in **Figure 7**, we also evaluated the subjective appetite and sleepiness level by VAS. Significant interactions between time and groups on hunger, fullness was observed in stomach fullness, desire to eat, and capacity to eat were showed in the two-way repeated ANOVA model (**Supplementary Table 8**). While no statistical differences in five appetite indicators were observed at the timepoints of pre-prandial before each meals and 2-hour after the first meal, TRF group exhibited a significant lower score in hunger, desire to eat, and capacity to eat levels (all p ≤ 0.026). Accordingly, the scores of fullness and stomach fullness levels were significantly higher before the third meal in the TRF group (all p ≤ 0.005). It is worth noting that, at the timepoint of before sleep, TRF substantially increased the score of hunger (Δ = 15.91 ± 4.66 mm, p=0.006), desire to eat (Δ = 24.45 ± 3.29 mm, p < 0.001), and capacity to eat (Δ = 13.45 ± 3.49 mm, p=0.003), as well as reduced the fullness level (Δ = -15.18 ± 4.49 mm, p=0.007). However, there were not any significant differences in subjective appetite between groups the next day morning. What’s more, subjective sleepiness level was unaffected by TRF intervention.

## Discussion

An increasing number of studies have demonstrated that TRF intervention can produce 1-4% weight loss over 1 to 16 weeks. Although current available data did not support a stronger effect of TRF on body weight and metabolic improvement as compared to continuous caloric restriction, the mechanistic explanation behind this phenomenon remains raised great interest to researchers. Some controversial issues, including inaccurate recording of food intake, unmonitored energy expenditure and the involvement of physiological adaptation were still need to be determined (8–11, 13, 14, 21–24, 26, 32, 33). Therefore, we conducted the first rigorous trial to systematically quantify and compare the energy balance during TRF intervention in healthy subjects. We strictly controlled and ensured that energy intake, fluid intake, and lifestyle were consistent during the interventions except for eating schedules, while energy expenditure was monitored using metabolic chamber and all energy excretion including feces and urine were measured by bomb calorimeter. Surprisingly, TRF could evoke a significant fecal energy loss and a trend in urine energy loss without energy expenditure alteration, which caused a negative energy balance, while 24-hour blood glucose and heart rate were also improved during TRF. Our findings are consistent with the benefits of long-term TRF intervention reported previously, supporting the use of TRF as an alternative dietary strategy for obesity.

In this study, we found no significant differences in total energy expenditure during the whole intervention period. Despite that some studies suggested that TRF induced adiponectin elevation might contribute to increased energy expenditure (10, 25, 34), the results of the current study are consistent with previous studies: through 24-hour energy monitoring or resting metabolic rate measurements, there is no convincing evidence in support of increased energy expenditure due to TRF (20, 26, 35). But several components of total energy expenditure are inconsistent with a prior study, including higher TEF, increased diurnal energy expenditure, and decreased nocturnal energy expenditure in TRF group (20). These negligible differences might be explained by different food macronutrient compositions and time schedules among two studies, respectively.

About 2-10% of food consumed ends up being excreted in feces. Even if the nutrients enter the circulation, there is still 1-2% of total energy intake finally being filtered by the kidneys (36). The precise measurement of the third and often neglected factor, energy excretion finally helped reveal that TRF has resulted in a negative energy balance of -46 Kcal, which is equivalent to -2.6% of total energy intake, as a result of increasing fecal (by nearly 22.7%) and urine (by nearly 14.5%) energy loss. Recently, Balso et al. showed that relative fecal energy loss is on average 6% during overfeeding and 9% during underfeeding, further confirming the variability of energy excretion (29). The magnitude of 2.6% energy losing effect could be translated to 0.7 Kg body weight loss in an 83.9 Kg subject over 8 weeks (19). In Moro et al. study, 8-week TRF under isocaloric condition led to average 0.97 Kg weight loss in 17 healthy subjects and our data indicated that TRF induced negative energy balance might explain approximately 72.2% of their weight loss in their long-term intervention (10). In McAllister et al. study, isocaloric treatment in TRF group showed 0.6kg weight loss after 4 weeks of intervention. The –2.6% energy losing effect could be translated to 0.4Kg body weight loss during the same condition, which explained nearly 66.7% of the weight loss (26). In Kahleova et al. study, participants underwent the same calorie restriction amount but with different intervention regimens, either six or two meals a day. After 12 weeks of intervention, body weight decreased (1.4 kg) more in two meals group (breakfast and lunch) than six meals group, although dietary intake, step count, and REE showed no differences. Here, our model could be translated to 0.9kg body weight loss, which explained 64.3% of the total difference (37). Tiny deviations from energy balance, on the order of 1-2% of daily energy intake, can indeed result in considerable long-term changes in body weight (~20 kg) (38). Therefore, the potential long-term implications of such a negative energy balance must not be underestimated. The current energy balance model still cannot explain a small part of weight loss. Therefore, it is undoubtedly necessary to directly evaluate the long-term impact of TRF on energy balance.

Metabolic flexibility represents the capacity of body to adapted nutrient challenge from environment. Our results, consistent with the previous study, suggested that TRF intervention significantly enhanced metabolic flexibility (20), but this result should be interpreted with caution, since the dinner in the control group should largely affected the nighttime RQ, such substantial alteration in the delta npRQ might be caused by the imbalance eating schedule, rather than the equal-proportion improvement of adaptation ability (39).Contrary to the previous study, we found that the substrate balance significantly shifted towards carbohydrate in TRF group and npRQ increased accordingly (20). These conflicts may induce by the different experiment schedules. It is also possible that different food compositions and healthy or overweight participants may account for the conflicts. Future investigations including acute and chronic effects of TRF on RQ are encouraged.

Hypoglycemic effect of TRF has also been proved, as in line with previous studies (10, 14, 17). The average glucose over 24-hour TRF intervention was reduced by 5%, which was largely contributed by the extensive attenuation of postprandial glycemic profiles in TRF group. The postprandial insulin secretion was also reduced in TRF group. Therefore, it is unlikely to associate the hypoglycemic effect of TRF with extra insulin secretion. It should be noted that, FFA is a well-known marker of insulin resistance, it could blunt the hypoglycemic effect of insulin by increasing beta-oxidation in the mitochondrial. Thus, as the pre-prandial FFA substantially reduced before 2nd and 3rd meal during TRF, subjects might stay in a more insulin-sensitized status, which explained the simultaneously reduction of postprandial glucose and insulin. Moreover, the FFA levels were constant lower during eating period in TRF group, suggesting a remarkable improvement of lipotoxicity over 24 hours. This might explain the better insulin sensitivity after long-term TRF intervention. Consistently our linear mixed-effects model on postprandial glucose also emphasized the importance of pre-prandial status in explaining the improvement of glycemic profiles during TRF. This model suggested an independent contribution of pre-prandial insulin to TRF-induced glycemic improvement. However, there is still a large part of TRF effect that could not be explained, which deserves to be further studied. Additionally, attenuation of postprandial glycemic responses in TRF group resulted in a remarkable attenuation of glycemic variability over 24 hours, MAGE and glycemic CV were significantly reduced by 26% and 31% respectively over 24-hour TRF intervention. Since glycemic variability has been demonstrated to be closely correlated to oxidative stress and beta-cell function in previous studies (39), such extensive attenuation of glucose fluctuation may help to explain the long-term benefits of TRF for glycemic control (40).

As regarded to the physiological profiles during TRF, we observed a paralleling tendency of energy expenditure, heart rate,and respiratory rate during 11:00 to 13:00 and 19:00 to 21:00, strongly suggested a cardiovascular/respiratory-related thermogenic process, which would be inevitably reflected in the flux of body temperature, but we didn’t observe such a corresponding change in our data. This might due to the infrared sensors in this study, and the responsiveness of the sensor, as time constant, is about 90 minutes. This may largely blunt its sensitivity to skin temperature alternations. The hyperresponsiveness skin temperature and core temperature sensors are needed to capture subtle changes. We didn’t observe a decrease in blood pressure and even a temporal increase in systolic blood pressure during postprandial period at TRF 3^rd^ meal, which is inconsistent with previous data (24). Our participants were healthy subjects and only 24-hour TRF intervention could explain this disagreement. Nevertheless, we did observe the TRF significantly reduced heart rate at nocturnal and exercise periods. There is increasing evidence that increased heart rate is associated with hypertension and mortality, while decreased heart rate could help improve the situation (41).

We also investigated the effect of TRF on subjective appetite. Interestingly, although the self-reported appetite scores showed that subjects exhibited higher hunger before bedtime during TRF, such hunger went to equal between groups in the following morning. Since the importance of adherence to dietary recommendations in successful weight loss during long-term (42), this bearable hunger make TRF a more feasible way to control body weight. These improvements of appetite related factors was corroborated by other studies during TRF intervention (16, 20). Despite an overall positive attitude towards TRF eating pattern, this barrier to adopting the TRF eating pattern needs further investigation, as how to overcome the increased desire to eat before bedtime until the next morning.

To the best of our knowledge, we designed the most stringent control study to evaluate the actual effect of TRF on energy balance. Some limitations of the present study should be considered. First, we only observed the acute effect of TRF on energy balance and physiological-biochemical parameters, which may not be long enough to reach a new energy balance or metabolic adaptation. Second, Lowe et al. study found that late TRF, as ate ad libitum from 12:00 PM until 8:00 PM, didn’t reduce participants’ body weight after 12-week intervention (33). Here, this study only evaluated the effect of early TRF within a fixed time window, thus we were unable to answer whether the energy balance induced by TRF in different time windows shows rhythmic oscillations. Compare with caloric restriction, TRF produces less than 5% weight loss in the trails, which do not meet the threshold of clinically significant weight loss. Whether TRF plus mild caloric restriction produce a 1 + 1>2 effect? Long-term and well-designed trials are needed find out the optimal TRF strategy. Third, the participants recruited in this study were all young healthy adults. Chaix et al. have proved that 9-hour TRF benefits was sex- and age-dependent in mice (43). Thus, whether our finding is equally applicable to other populations including different ages, sexes, and disease states such as obesity and diabetes needs to be further investigated.

In conclusion, our study proved that TRF could induce negative energy balance by increasing fecal and urine energy excretion, and this seemingly negligible energy excretion elevation can explain up to 64.3% - 72.2% of weight loss in previous studies. As a result, the mystery of how TRF regulates energy balance might be unraveled, and hence provides a fundamental explanatory mechanism for weight loss. Our data also indicated an improvement in glycemic profiles, heart rate, respiration rate as well as metabolic flexibility during TRF. However, subjective appetite increased prior to bedtime might hinder the execution of TRF strategy in the long term. In light of these promising results, future research is needed to better elucidate the long-term metabolic adaptation of TRF, including the ketone body concentration and interconversion among macronutrients. On the other hand, more randomize controlled studies with well-designed and diverse population are also needed.

## Data Availability Statement

The raw data supporting the conclusions of this article will be made available by the authors, without undue reservation.

## Ethics Statement

The studies involving human participants were reviewed and approved by Ethics committee of Ruijin Hospital affiliated to Shanghai Jiaotong University. The patients/participants provided their written informed consent to participate in this study.

## Author Contributions

RB and YS contributed to project management, participants recruitment, data collection, and data analysis. YJ and LY contributed to acquiring ethical approval for this trial. JH and WW contributed to acquiring funding for this trial and oversaw the design, and execution for this study. All authors contributed to the composition and revision of the manuscript and gave final approval of its content.

## Funding

This study was sponsored by the grants from the National Science Foundation of China (KY2019338).

## Conflict of Interest

The authors declare that the research was conducted in the absence of any commercial or financial relationships that could be construed as a potential conflict of interest.

## Publisher’s Note

All claims expressed in this article are solely those of the authors and do not necessarily represent those of their affiliated organizations, or those of the publisher, the editors and the reviewers. Any product that may be evaluated in this article, or claim that may be made by its manufacturer, is not guaranteed or endorsed by the publisher.

## Supplementary Material

Supplementary Figure 1 – Study Design. **(A)** The overall study design over 3 days. **(B)** The schedule of daily activity during the interventions.
