# Supplementary material for: Effects of Time-Restricted Feeding on Energy Balance: A Cross-Over Trial in Healthy Subjects
Source: Front Endocrinol (Lausanne). 2022 Apr 27;13:870054. doi: 10.3389/fendo.2022.870054 (PMC9092453; doi:10.3389/fendo.2022.870054)
Supplement: Supplementary file 2 [file Table_1.docx]

| **Supplementary Table 1 - Food energy and macronutrients composition** | | | |
| --- | --- | --- | --- |
|  | **Food with brilliant blue** | **Food with carmine** | **p Value** |
| Energy density (kcal/g) | 4.69 ± 0.03 | 4.67 ± 0.01 | 0.666 |
| Carbohydrate (%) | 56.55 ± 0.83 | 56.62 ± 1.07 | 0.965 |
| Fat (%) | 29.74 ± 1.06 | 29.56 ± 1.03 | 0.916 |
| Protein (%) | 13.71 ± 0.23 | 13.82 ± 0.04 | 0.720 |
| *Data were presented as mean ± SEM.  *Differences between group were tested by Student's t test. | | | |
