# Supplementary material for: Effects of Time-Restricted Feeding on Energy Balance: A Cross-Over Trial in Healthy Subjects
Source: Front Endocrinol (Lausanne). 2022 Apr 27;13:870054. doi: 10.3389/fendo.2022.870054 (PMC9092453; doi:10.3389/fendo.2022.870054)
Supplement: Supplementary file 3 [file Table_2.docx]

| **Supplementary Table** **2 - Two-way Repeated Measurements ANOVA Model with Metabolic Parameter as Dependent Variable** | | | | | |
| --- | --- | --- | --- | --- | --- |
| **Source** | **Effect Size** | **DFn** | **DFd** | **F** | **p** |
| **Dependent Variable: Hourly Energy Expenditure** | | | | | |
| Group | 0.008 | 1 | 11 | 0.091 | 0.768 |
| Duration | 0.931 | 23 | 253 | 147.901 | <0.001 |
| Group: Duration | 0.203 | 23 | 253 | 2.804 | <0.001 |
| **Dependent Variable: Hourly non-protein RQ** | | | | | |
| Group | 0.283 | 1 | 11 | 4.335 | 0.061 |
| Duration | 0.637 | 23 | 253 | 19.266 | <0.001 |
| Group: Duration | 0.375 | 23 | 253 | 6.603 | <0.001 |

*Effect Size represents the partial eta squared of model

*P values were calculated by pairwise t-test with Holm–Bonferroni adjustment
