# Supplementary material for: Effects of Time-Restricted Feeding on Energy Balance: A Cross-Over Trial in Healthy Subjects
Source: Front Endocrinol (Lausanne). 2022 Apr 27;13:870054. doi: 10.3389/fendo.2022.870054 (PMC9092453; doi:10.3389/fendo.2022.870054)
Supplement: Supplementary file 4 [file Table_3.docx]

| **Supplementary Table 3 - 24-Hour CMG data** | | | | |
| --- | --- | --- | --- | --- |
|  | **Control** | **TRF** | **Percent change** | **p Value** |
| 24-hour average blood glucose (mmol/L) | 5.61 ± 0.12 | 5.34 ± 0.07 | -5% | 0.007 |
| Diurnal glucose (mmol/L) | 6.00 ± 0.16 | 5.67 ± 0.10 | -6% | 0.001 |
| Nocturnal glucose (mmol/L) | 4.98 ± 0.12 | 4.93 ± 0.08 | -1% | 0.740 |
| Coefficient of variation (%) | 0.22 ± 0.02 | 0.16 ± 0.01 | -26% | 0.007 |
| LAGE (mmol/L) | 5.76 ± 0.39 | 4.70 ± 0.37 | -18% | 0.080 |
| MAGE (mmol/L) | 3.72 ± 0.35 | 2.56 ± 0.27 | -31% | 0.014 |
| *Data were presented as mean ± SEM.  *Differences between group were tested by pairwise t-test with Holm–Bonferroni adjustment  *The percentage change was calculated by dividing the differences of mean between TRF and control groups.  *CGM data were obtained from Dexcom G5. | | | | |
