# Supplementary material for: Effects of Time-Restricted Feeding on Energy Balance: A Cross-Over Trial in Healthy Subjects
Source: Front Endocrinol (Lausanne). 2022 Apr 27;13:870054. doi: 10.3389/fendo.2022.870054 (PMC9092453; doi:10.3389/fendo.2022.870054)
Supplement: Supplementary file 5 [file Table_4.docx]

| **Supplementary Table 4 - Postprandial glycemic and insulin profiles of each meal** | | | | | | | |
| --- | --- | --- | --- | --- | --- | --- | --- |
|  | **Glucose (mmol/L)** | | |  | **Insulin (*µ*IU/ml)** | | |
|  | **Control** | **TRE** | **P** |  | **Control** | **TRE** | **P** |
| **1st Meal** | | | | | | | |
| Pre-prandial | 5.13 ± 0.11 | 4.95 ± 0.13 | 0.262 |  | 6.39 ± 0.75 | 5.62 ± 0.53 | 0.257 |
| 0.5-hr Postprandial | 6.23 ± 0.13 | 6.33 ± 0.19 | 0.196 |  | 70.43 ± 10.14 | 87.56 ± 12.99 | 0.326 |
| 1-hr Postprandial | 7.75 ± 0.38 | 8.06 ± 0.45 | 0.701 |  | 85.53 ± 8.27 | 77.84 ± 11.76 | 0.407 |
| 2-hr Postprandial | 5.81 ± 0.25 | 5.89 ± 0.21 | 0.89 |  | 38.6 ± 5.06 | 38.4 ± 5.17 | 0.979 |
| 2-hr AUC (Absolute) | 13.05 ± 0.37 | 13.07 ± 0.43 | 0.968 |  | 119.62 ± 10.48 | 120.23 ± 9.56 | 0.931 |
| 2-hr AUC (Incremental) | 2.78 ± 0.36 | 3.17 ± 0.36 | 0.432 |  | 106.95 ± 9.81 | 109.08 ± 9.18 | 0.786 |
| **2nd Meal** | | | | | | | |
| Pre-prandial | 5.02 ± 0.09 | 4.69 ± 0.17 | 0.094 |  | 4.06 ± 0.44 | 19.7 ± 2.95 | <0.001 |
| 0.5-hr Postprandial | 6.10 ± 0.28 | 5.94 ± 0.19 | 0.952 |  | 69.25 ± 19.03 | 63.45 ± 8.65 | 0.69 |
| 1-hr Postprandial | 9.22 ± 0.49 | 7.09 ± 0.21 | <0.001 |  | 86.92 ± 15.15 | 66.32 ± 6.75 | 0.131 |
| 2-hr Postprandial | 7.00 ± 0.31 | 5.79 ± 0.18 | 0.001 |  | 55.54 ± 8.66 | 29.02 ± 4.96 | 0.008 |
| 2-hr AUC (Absolute) | 14.80 ± 0.61 | 12.31 ± 0.28 | <0.001 |  | 127.98 ± 21.93 | 100.2 ± 8.21 | 0.171 |
| 2-hr AUC (Incremental) | 4.75 ± 0.56 | 2.93 ± 0.23 | 0.0055 |  | 119.93 ± 22.07 | 61.14 ± 5.16 | 0.012 |
| **3rd Meal** | | | | | | | |
| Pre-prandial | 4.69 ± 0.08 | 5.57 ± 0.14 | 0.004 |  | 3.98 ± 0.43 | 22.11 ± 4.65 | 0.002 |
| 0.5-hr Postprandial | 6.05 ± 0.12 | 6.09 ± 0.17 | 0.997 |  | 58.51 ± 13.28 | 46.22 ± 6.2 | 0.24 |
| 1-hr Postprandial | 8.84 ± 0.36 | 7.05 ± 0.32 | <0.001 |  | 78.59 ± 13.43 | 59.47 ± 5.98 | 0.143 |
| 2-hr Postprandial | 7.22 ± 0.25 | 6.12 ± 0.20 | 0.005 |  | 60.16 ± 10.08 | 33.29 ± 5.05 | 0.018 |
| 2-hr AUC (Absolute) | 14.83 ± 0.47 | 12.57 ± 0.33 | <0.001 |  | 118.75 ± 18.67 | 89.32 ± 9.18 | 0.085 |
| 2-hr AUC (Incremental) | 4.92 ± 0.55 | 1.42 ± 0.35 | <0.001 |  | 110.87 ± 18.48 | 45.47 ± 9.87 | 0.004 |
| *Data were presented as mean ± SEM.  *Differences between group were tested by pairwise t-test with Holm–Bonferroni adjustment  *AUCs were calculated by trapezoidal rule | | | | | | | |
