# Supplementary material for: Effects of Time-Restricted Feeding on Energy Balance: A Cross-Over Trial in Healthy Subjects
Source: Front Endocrinol (Lausanne). 2022 Apr 27;13:870054. doi: 10.3389/fendo.2022.870054 (PMC9092453; doi:10.3389/fendo.2022.870054)
Supplement: Supplementary file 6 [file Table_5.docx]

| **Supplementary Table 5 - Linear mixed-effects models to evaluate the effects of metabolic components on TRF-induced postprandial glycemic response** | | | | | | | |
| --- | --- | --- | --- | --- | --- | --- | --- |
| **Effects** | **Estimate** | **SE** | **DF** | **T value** | **P value** | **95% CI** | |
|  |  |  |  |  |  | **Lower** | **Upper** |
| **Model 1** | | | | | |  |  |
| (Intercept) | 247.489 | 18.909 | 35 | 13.088 | <0.001 | 210.427 | 284.551 |
| TRF intervention | -93.19 | 17.345 | 34 | -5.373 | <0.001 | -127.186 | -59.194 |
| Pre-prandial glucose | -104.641 | 19.152 | 34 | -5.464 | <0.001 | -142.179 | -67.103 |
| **Model 2** | | | | | |  |  |
| (Intercept) | 725.533 | 99.201 | 32 | 7.314 | <0.001 | 531.099 | 919.967 |
| TRE intervention | -68.279 | 20.428 | 32 | -3.342 | 0.002 | -108.318 | -28.24 |
| Carbohydrate oxidation | -1.637 | 1.27 | 32 | -1.289 | 0.207 | -4.126 | 0.852 |
| Pre-prandial insulin | -2.55 | 1.046 | 32 | -2.112 | 0.015 | -6.26 | -1.16 |
| Pre-prandial glucose | -86.73 | 20.019 | 32 | -4.332 | <0.001 | -125.967 | -47.493 |
| *Dependent variable of model 1 and model 2 was the incremental AUCs of postprandial glucose  *The column of “Effect” represented the independent variables in model 1 and model 2  *Both models use the subjects and meals as random effects  *The column of “Estimate” represented the effect of each independent variable | | | | | | | |
