# Supplementary material for: Effects of Time-Restricted Feeding on Energy Balance: A Cross-Over Trial in Healthy Subjects
Source: Front Endocrinol (Lausanne). 2022 Apr 27;13:870054. doi: 10.3389/fendo.2022.870054 (PMC9092453; doi:10.3389/fendo.2022.870054)
Supplement: Supplementary file 7 [file Table_6.docx]

| **Supplementary Table 6 - Pre and postprandial FFA of each meal** | | | |
| --- | --- | --- | --- |
|  | **Control** | **TRF** | **p** |
| **1st Meal** |  |  |  |
| Pre-prandial | 0.53 ± 0.2 | 0.53 ± 0.15 | 0.976 |
| 0.5h-postprandial | 0.32 ± 0.14 | 0.32 ± 0.09 | 0.873 |
| 1h-postprandial | 0.14 ± 0.05 | 0.11 ± 0.04 | 0.175 |
| 2h-postprandial | 0.15 ± 0.04 | 0.15 ± 0.05 | 0.802 |
| **2nd Meal** |  |  |  |
| Pre-prandial | 0.59 ± 0.13 | 0.21 ± 0.05 | <0.001 |
| 0.5h-postprandial | 0.6 ± 0.14 | 0.21 ± 0.05 | <0.001 |
| 1h-postprandial | 0.26 ± 0.07 | 0.16 ± 0.03 | 0.001 |
| 2h-postprandial | 0.17 ± 0.06 | 0.16 ± 0.04 | 0.428 |
| **3rd Meal** |  |  |  |
| Pre-prandial | 0.7 ± 0.19 | 0.19 ± 0.04 | <0.001 |
| 0.5h-postprandial | 0.67 ± 0.22 | 0.18 ± 0.06 | <0.001 |
| 1h-postprandial | 0.26 ± 0.11 | 0.14 ± 0.03 | 0.002 |
| 2h-postprandial | 0.15 ± 0.03 | 0.14 ± 0.04 | 0.206 |

*Data were presented as mean ± SEM.

*Differences between groups were tested by pairwise t-test with Holm–Bonferroni adjustment.
