# Supplementary material for: Effects of Time-Restricted Feeding on Energy Balance: A Cross-Over Trial in Healthy Subjects
Source: Front Endocrinol (Lausanne). 2022 Apr 27;13:870054. doi: 10.3389/fendo.2022.870054 (PMC9092453; doi:10.3389/fendo.2022.870054)
Supplement: Supplementary file 8 [file Table_7.docx]

| **Supplementary Table 7 - Two-way Repeated Measurements ANOVA Model with Physiological Parameter as Dependent Variable** | | | | | |
| --- | --- | --- | --- | --- | --- |
| **Source** | **Effect Size** | **DFn** | **DFd** | **F** | **p** |
| **Dependent Variable: Hourly Average Heart Rate** | | | | | |
| Group | 0.167 | 1 | 10 | 2.01 | 0.187 |
| Duration | 0.921 | 23 | 230 | 116.116 | <0.001 |
| Group: Duration | 0.526 | 23 | 230 | 11.109 | <0.001 |
| **Dependent Variable: Hourly Average Respiratory Rate** | | | | | |
| Group | 0.118 | 1 | 10 | 1.344 | 0.273 |
| Duration | 0.826 | 23 | 230 | 47.364 | <0.001 |
| Group: Duration | 0.16 | 23 | 230 | 1.906 | 0.009 |
| **Dependent Variable: Hourly Average Systolic BP** | | | | | |
| Group | 0.019 | 1 | 9 | 0.171 | 0.689 |
| Duration | 0.666 | 23 | 207 | 17.923 | <0.001 |
| Group: Duration | 0.115 | 23 | 207 | 1.165 | 0.28 |
| **Dependent Variable: Hourly Average Diastolic BP** | | | | | |
| Group | 0.014 | 1 | 9 | 0.125 | 0.732 |
| Duration | 0.728 | 23 | 207 | 24.096 | <0.001 |
| Group: Duration | 0.12 | 23 | 207 | 1.227 | 0.225 |
| **Dependent Variable: Hourly Body Temperature** | | | | | |
| Group | 0.146 | 1 | 8 | 1.369 | 0.276 |
| Duration | 0.837 | 23 | 184 | 41.028 | <0.001 |
| Group: Duration | 0.122 | 23 | 184 | 1.112 | 0.336 |

*Effect Size represents the partial eta squared of model

*P values were calculated by pairwise t-test with Holm–Bonferroni adjustment
