# Supplementary material for: Effects of Time-Restricted Feeding on Energy Balance: A Cross-Over Trial in Healthy Subjects
Source: Front Endocrinol (Lausanne). 2022 Apr 27;13:870054. doi: 10.3389/fendo.2022.870054 (PMC9092453; doi:10.3389/fendo.2022.870054)
Supplement: Supplementary file 9 [file Table_8.docx]

| **Supplementary Table 8 - Two-way Repeated Measurements ANOVA Model with VAS Score as Dependent Variable** | | | | | |
| --- | --- | --- | --- | --- | --- |
| **Source** | **Effect Size** | **DFn** | **DFd** | **F** | **p** |
| **Dependent Variable: VAS Score of Hunger** | | | | | |
| Group | 0.632 | 1 | 9 | 15.467 | 0.003 |
| Time | 0.744 | 7 | 63 | 26.106 | <0.001 |
| Group: Time | 0.561 | 7 | 63 | 11.496 | <0.001 |
| **Dependent Variable: VAS Score of Fullness** | | | | | |
| Group | 0.507 | 1 | 9 | 9.255 | 0.014 |
| Time | 0.632 | 7 | 63 | 15.459 | <0.001 |
| Group: Time | 0.371 | 7 | 63 | 5.313 | <0.001 |
| **Dependent Variable: VAS Score of Stomach Fullness** | | | | | |
| Group | 0.402 | 1 | 9 | 6.042 | 0.036 |
| Time | 0.501 | 7 | 63 | 9.023 | <0.001 |
| Group: Time | 0.352 | 7 | 63 | 4.896 | <0.001 |
| **Dependent Variable: VAS Score of Desire to Eat** | | | | | |
| Group | 0.449 | 1 | 9 | 7.325 | 0.024 |
| Time | 0.625 | 7 | 63 | 15.032 | <0.001 |
| Group: Time | 0.621 | 7 | 63 | 14.72 | <0.001 |
| **Dependent Variable: VAS Score of Capacity to Eat** | | | | | |
| Group | 0.076 | 1 | 9 | 0.741 | 0.412 |
| Time | 0.655 | 7 | 63 | 17.109 | <0.001 |
| Group: Time | 0.519 | 7 | 63 | 9.698 | <0.001 |

*Effect Size represents the partial eta squared of model

*P values were calculated by pairwise t-test with Holm–Bonferroni adjustment
